# The University of Maryland Dental Department and the Secretary of the National Association of Dental Examiners

**Published:** 1888-12

**Authors:** 


### Editorial, Etc.

THE UNIVERSITY OF MARYLAND DENTAL DEPARTMENT AND THE SECRETARY OF THE NATIONAL ASSOCIATION OF DENTAL EXAMINERS.—

At the request of Dr. Fred. A. Levy, Sec'y. "National Association of Dental Examiners," we publish the following letter in answer to one from the Dean of the Md. Univ. Dental Department, dated Nov. 1st, 1888, and which appeared in the Oct. No. of this JOURNAL:

ORANGE, N. J., November 5th, 1888.

*Ferdinand J. S. Gorgas, M. D., D. D. S., Dean, Baltimore, Md.*

*Dear Sir.*—Yours of November 1st, enclosing Circulars containing correspondence with Drs. Waters, Wright and Wardlaw, received, and in reply I would state:

1st. That I have no record of the attendance of Dr. Wardlaw and it appears from his own statement that he was not present at the Session at which was passed the Resolution complained of by you.

2nd. Dr. Wright is in error relative to the proceedings of the National Association of Dental Examiners when he states that your College passed without objection or comment of any kind. In this important particular: that there was opposition to the insertion of the name of your College among those whose diplomas to the National Association of Dental Examiners recommended the various State Boards to accept instead of an examination, or it would not have been dropped from the revised list presented by the Committee; for particulars of the passage of this resolution I refer you to page 4 of the printed proceedings of the Association, from which it appears that the list of Colleges presented and accepted at the last meeting was re-considered. The list was then referred back to the Committee to be revised, and was again presented to the Association; was amended, and accepted omitting the name of your College.

3rd. That this *report* of the committee, appointed for that purpose, consisting of Drs. Wright, Rawls and Levy, was *signed* by each of them, and in that form, presented to the Association; this *original* report, omitting the name of your College, and signed by Dr. Wright with the others mentioned, I have in my possession, which you can see upon application.

4th. At the time of the passage of this resolution, as appears upon page 4 of the proceedings mentioned, the roll-call showed ten States represented.

5th. The printed report of the proceedings of the Association sent you are correct in every particular, the assertion of Dr. Wardlaw (who was not present), and those of Dr. Wright (who concurred in the resolution dropping your College), to the contrary notwithstanding. As you will see, the conclusions which you arrive at, from what you choose to term the testimony in your possession, are totally incorrect, inasmuch as the name of your College was intentionally omitted from the list of the Dental Colleges which the National Association recommended the various State Boards, whose diplomas might be accepted, instead of an examination.

There has been no confusion upon my part in the matter; upon the contrary, I have been exceedingly particular, and know that there has been no mistake whatever.

Any other or further information you may desire I shall be pleased to furnish you.

Very truly yours,
FRED. A. LEVY,
Secretary.

In answer to the above letter of Dr. Levy and especially the portion of it relating to the action of Dr. G. F. S. Wright, President of the South Carolina State Board of Dental Examiners, we refer to a letter of Dr. Wright's dated Nov. 15th, 1888, which was published in the November No. of this JOURNAL, and which we again present to our readers:

" GEORGETOWN, S. C., Nov. 15th, 1888.

*Professor F. J. S. Gorgas, Dean of Dental Department University of Maryland :*

*Dear Doctor.*—The Committee of which Dr. Levy writes, whose report he says I signed, held no meeting when I was present, and the final action was as I stated.

The roll was called, and as the Secretary called the name of an institution, the Presiding Officer, Dr. Waters, would ask if there was any objection, and if there was nothing said he would announce: "*No objection.*" I kept a score of the roll, and no one objected to the University of Maryland Dental Department. I have written this to Dr. Waters, who has written to me and stated his impression of the action. Dr. Levy says the proceedings will be published in the *Ind.* I am not in possession of any information except rumors from the Association of Dental Faculties; of these the impression made was not unfavorable to the School of which you are the Dean.

That the Dental Department of the University of Maryland was objected to is a mistake, it was not. No school in

Baltimore was, but a Washington School did have objection made and was turned down.

I am very respectfully,

G. F. S. WRIGHT, D. D. S.

President of S. C. State Dental Examining Board."

It therefore becomes a question of veracity between the two gentlemen, Drs. Wright and Levy. Dr. W. C. Wardlaw, of the Georgia State Board of Dental Examiners, supports Dr. Wright's statement so far as the following declaration shows:

"I, with Dr. S. B. Barfield, of Macon, Ga., represented the Georgia Examining Board in the Louisville meeting, and my recollection is clear upon this matter. For having heard whisperings that objections might be made against your College, I, at considerable inconvenience, attended the Session of The National Board of Dental Examiners, at which the roll of Colleges was reviewed, prepared to fight the question, and was consequently upon the *qui vive*. The roll had been gone over at a previous meeting, and in being reviewed for adoption at this session, was gone through with two or three times, and each time there were errors to be corrected, so that I am not surprised if the mistake (unintentional) does actually exist in the Secretary's record. But a mistake it certainly is. I feel sure that Dr. Barfield and others will substantiate me in this.

There was one subsequent session of the Board of Dental Examiners at which I was not present, but at which I was told, nothing had been done but the election of officers, and routine business.

I voluntarily write this, so that if the records of Secretary Levy are wrong, your college may still be set right."

Assuming that Dr. Levy's statement and minutes of the proceedings of Louisville meeting are correct, we contend that the action of the "National Board of Dental Examiners," at the Louisville meeting, in omitting the name of the University of Maryland Dental Department, was contrary to a standing resolution of said association which recommends to all State Boards to confer with a college whose diplomas are questioned before publishing it as derelict, to the end that no injustice may be done, particularly to those who have honestly earned their diplomas from such institution.

The spirit and the letter of this resolution is set at nought if the president and the secretary of said Association are correct in their reports of what took place in regard to the University of Maryland Dental Department, and Dr. Gorgas in his letter to Dr. Waters very justly asks:

"Have there ever been any charges brought against our Dental Department for failure to comply with any of the requirements of the State Boards of Dental Examiners, or of the American and Southern Dental Associations? I have never heard of any, and defy any one to prove that we have not strictly complied with all such requirements.

*Does not the fact that no less than three of the five members composing the Maryland Board of Dental Examiners have recommended students to our Dental Department for the present session,* prove that the maj rity of the members of the Maryland Board of Dental Examiners, are satisfied with our course of instr ction and recommend it?"

Again. Article III of the Constitution of the National Association of Dental Examiners reads:

"MEMBERS.—This Ass ciation shall consist of such different State Boards of Dental Examiners as may elect to join this National Association. They may be represented either by a delegate or delegates duly authorized, or by the whole Board. Certificates from the proper officers of the Board will be necessary to entitle such Board to representation in this body"

Dr. Waters was not duly authorized to represent the Maryland State Board of Dental Examiners at the Louisville meeting. He could not therefore and in point of fact did not present a "certificate from the proper officers" and every member of the Maryland Board of Dental Examiners, except Dr. Waters, has said individually that Dr. Waters was not an accredited representative of the Maryland Dental Examiners to Louisville. When the president of the board went to the meeting at Niagara in 1884 and when the secretary went to the meeting at New Orleans in 1885, they were duly commissioned under seal and money appropriated to meet their expenses (in part).

The interests involved and the gravity of the situation, made it necessary to present the correspondence which appears in this JOURNAL.

The remark of a delegate at a meeting of the National Association of Dental Examiners, that "he was glad to see the quarrel between the Baltimore colleges" illustrates the "true inwardness" of the effort to depreciate and ostracize the University of Maryland Dental Department, although in the suitably expressive parlance of Dr. Waters that "school is not the only one left out in the cold."

The President of the Maryland State Board of Dental Examiners, Dr. E. P. Keech, and the secretary, Dr. S. M. Field, have both recently stated that they would not discriminate between graduates of the University of Maryland Dental Department and those of other dental schools.

The Maryland Dental Law originally denied recognition to diplomas granted in other States, but amendments were adopted which placed it in harmony with other dental laws. Formerly a person who held a diploma from a university or a college of Maryland authorized to grant diplomas in dental surgery could require his name to be registered; now the board has discretionary power to pass upon the qualifications of any one who desires to begin the practice of dentistry in Maryland. Although the dental law of 1884 fully protected the University of Maryland Dental Department in this State from false friends or open enemies yet its dean in order to assist in removing the restrictive feature of the law favored the amendments referred to and wrote: "I heartily approve of them and desire to see the bill passed by the Legislature."

As an evidence of the desire of the Faculty of the University of Maryland Dental Department to adhere strictly to the recommendations of the State Boards of Dental Exaniners, the American Dental Association, &c., reference can be made to the fact that during the session of 1885 86, no less than seventeen applicants for admission to the senior or graduating class were refused on the ground that five or more years practice were not equivalent to one session's attendance; and this was at least one year before the other dental schools pursued the same course.

We also desire to state that Dr. Levy's letter would have appeared in the preceding No. of this JOURNAL, but for the reason that the original was sent South to parties whose veracity it questioned; and as soon as a copy was sent to us by Dr. Levy, it was at once placed in the printer's hands for the present No. of JOURNAL. There was no desire on our part to suppress the letter as Dr. Levy intimates in his article in the *Western Dental Journal.*

